# Fuzzy C-Means Clustering: A Review of Applications in Breast Cancer Detection

**DOI:** 10.3390/e25071021

**Published:** 2023-07-04

**Authors:** Daniel Krasnov, Dresya Davis, Keiran Malott, Yiting Chen, Xiaoping Shi, Augustine Wong

**Affiliations:** 1 Department of Computer Science, Mathematics, Physics and Statistics, University of British Columbia, Kelowna, BC V1V 1V7, Canada; dkrasnov@student.ubc.ca (D.K.); kmalott@student.ubc.ca (K.M.); yiting_chen_2@sfu.ca (Y.C.); 2 Faculty of Health and Social Development, School of Nursing, University of British Columbia, Kelowna, BC V1V 1V7, Canada; dresya@student.ubc.ca; 3 Department of Statistics and Actuarial Science, Simon Fraser University, Burnaby, BC V5A 1S6, Canada; 4 Department of Mathematics and Statistics, York University, 4700 Keele Street, Toronto, ON M3J 1P3, Canada; august@yorku.ca

**Keywords:** biogeography-based optimization algorithm, firefly algorithm, fuzzy c-means clustering, genetic algorithm, image segmentation, mammogram

## Abstract

This paper reviews the potential use of fuzzy c-means clustering (FCM) and explores modifications to the distance function and centroid initialization methods to enhance image segmentation. The application of interest in the paper is the segmentation of breast tumours in mammograms. Breast cancer is the second leading cause of cancer deaths in Canadian women. Early detection reduces treatment costs and offers a favourable prognosis for patients. Classical methods, like mammograms, rely on radiologists to detect cancerous tumours, which introduces the potential for human error in cancer detection. Classical methods are labour-intensive, and, hence, expensive in terms of healthcare resources. Recent research supplements classical methods with automated mammogram analysis. The basic FCM method relies upon the Euclidean distance, which is not optimal for measuring non-spherical structures. To address these limitations, we review the implementation of a Mahalanobis-distance-based FCM (FCM-M). The three objectives of the paper are: (1) review FCM, FCM-M, and three centroid initialization algorithms in the literature, (2) illustrate the effectiveness of these algorithms in image segmentation, and (3) develop a Python package with the optimized algorithms to upload onto GitHub. Image analysis of the algorithms shows that using one of the three centroid initialization algorithms enhances the performance of FCM. FCM-M produced higher clustering accuracy and outlined the tumour structure better than basic FCM.

## 1. Introduction

Breast cancer is the second leading cause of cancer deaths in Canadian women [1]. Breast tumours are especially invasive due to their proximity to lymph nodes through which cancerous cells metastasize to distal sites [2]. Breast cancer survival rates increase with early detection by allowing patients to access a more diverse set of treatment options. This is of particular importance due to the idiopathic nature of breast cancers.

Classical methods, such as mammograms, detect breast cancer by relying on radiologists to recognize and coarsely outline the apparent non-benign lesions, and to highlight the size and location of the possible tumours [3]. The task is made difficult as abnormal lesions may present as masses of various sizes and borders or as microcalcifications indistinguishable by the naked eye. Radiologists, therefore, are not immune to under-reading, misreading, or missing presentations of small tumours due to noise in the mammogram [4,5]. The need for reliable interpretations thus necessitates radiologists to base their impression on multiple readings of one mammogram, making the task labour-intensive and cost-ineffective.

Computer-aided analyses are becoming increasingly prevalent in breast cancer identification. Newer methodologies to detect breast cancer supplement classical methods with automated mammogram analysis focused on highlighting malignant lesions for radiologists to interpret [6]. Most methods proposed are based on machine learning (ML), where algorithms are developed to automatically recognize patterns and trends in data without explicit programming [6,7]. A widely known algorithm used in image segmentation is fuzzy c-means (FCM) clustering [8,9]. FCM is an unsupervised machine learning clustering algorithm that computes the probability (membership value) of a certain data point—in this case, a pixel belonging to groups (cluster prototypes) consisting of points with significant similarities. The idea is similar to the expectation maximization (EM) algorithm in statistics, which is an iterative method that computes the posterior probability for each observation and allocates it to one of several possible groups so as to maximize the measured likelihood of a sample [10,11]. In FCM, clusters are modelled as circles. However, FCM is an iterative method dependent on the user-determined number of clusters and the random initialization of said clusters [12]. Both of these drawbacks may contribute to the algorithm converging to sub-optimal solutions [12,13,14]. The paper seeks to overcome these drawbacks by exploring three initialization algorithms to optimize FCM for breast cancer segmentation.

## 2. Background

### 2.1. Euclidean-Distance-Based Fuzzy C-Means Clustering

A widely known algorithm explored in image segmentation is fuzzy c-means (FCM) clustering. Introduced by Dunn in 1973 [15] and further iterated upon by Bezdek in 1981 [16], FCM clustering is a soft clustering algorithm that computes the probability (membership value) of a specific data point belonging to groups (cluster prototypes) consisting of points with significant similarities [9]. In the FCM algorithm, distance calculations are used to measure the similarity between data points to determine the probability that a data point belongs to a cluster. The traditional FCM algorithm is based on Euclidean distance. While Euclidean distance is optimized to detect spherical structural clusters, studies show that it does not compute accurate clustering with high dimensional data [17].

Let the Euclidean distance between two vectors $x={\left(x_{1},\ldots ,x_{d}\right)}^{⊤}$ and $y={\left(y_{1},\ldots ,y_{d}\right)}^{⊤}$ be:

$$d\left(x,y\right)=\sqrt{\sum_{p=1}^{d}{\left(x_{p}-y_{p}\right)}^{2}}$$

In performing fuzzy c-means clustering, the goal is to minimize the objective function:

$$J\left(U,C;X,m\right)=\sum_{i=1}^{c}\sum_{j=1}^{n}u_{ij}^{m}d_{ij}^{2}$$

subject to

(1) $$\begin{array}{cc} \sum_{i=1}^{c}u_{ij} & =1,\forall j\in \{1,\ldots ,n\} \end{array}$$

(2) $$\begin{array}{cc} \sum_{j=1}^{n}u_{ij} & >0,\forall i\in \{1,\ldots ,c\}, \end{array}$$

where

$$\begin{array}{cc} m & \;\mathrm{is}\;\mathrm{the}\;\mathrm{degree}\;\mathrm{of}\;\mathrm{fuzziness}\;(m>1),\\ X & =\{x_{1},\ldots ,x_{n}\}\;\mathrm{is}\;a\;\mathrm{set}\;\mathrm{of}\;\mathrm{data}\;\mathrm{points},\\ C & =\{c_{1},\ldots ,c_{c}\}\;\mathrm{is}\;\mathrm{the}\;\mathrm{set}\;\mathrm{of}\;\mathrm{cluster}\;\mathrm{prototypes}\;,\\ U & ={\left(u_{ij}\right)}_{c\times n}\;\mathrm{is}\;\mathrm{the}\;\mathrm{fuzzy}\;\mathrm{partition}\;\mathrm{matrix}\;,\\ d_{ij} & =d(c_{i},x_{j}). \end{array}$$

We apply the Lagrange multipliers method to solve the above optimization problem. Let $\lambda_{j}$, 0≤j≤n be the Lagrange multipliers in accordance with (1). Then, the Lagrangian is

$$L\left(U,C,\lambda ;X,m\right)=\sum_{i=1}^{c}\sum_{j=1}^{n}u_{ij}^{m}d_{ij}^{2}+\sum_{j=1}^{n}\lambda_{j}\left(1-\sum_{i=1}^{c}u_{ij}\right).$$

Minimizing the membership and the prototype yields the following optimal membership and cluster prototype update formula for the *i*th prototype and *j*th data point

(3) $$\begin{array}{cc} u_{ij} & =\frac{1}{\sum_{k=1}^{c}{\left(\frac{d_{ij}}{d_{kj}}\right)}^{\frac{2}{m-1}}} \end{array}$$

(4) $$\begin{array}{cc} c_{i} & =\frac{\sum_{j=1}^{n}u_{ij}^{m}x_{j}}{\sum_{j=1}^{n}u_{ij}^{m}} \end{array}$$

The pseudocode is shown in Algorithm 1 as follows [9]:
**Algorithm 1** FCM1:*C*: number of clusters2:*m*: the degree of fuzziness (m>1)3:ϵ: the error4:Initalize randomly the centers of clusters $c_{i}^{\left(0\right)}$5:Begin at iteration k=1.6:**repeat**7:     Calculate the membership $u_{ij}^{\left(k\right)}$ using the centers $c_{i}^{\left(k-1\right)}$:8:     $u_{ij}=\frac{1}{\sum_{k=1}^{c}{\left(\frac{d_{ij}}{d_{kj}}\right)}^{\frac{2}{m-1}}}$9:  Calculate the membership matrix $U^{\left(k\right)}={\left[u_{ij}\right]}_{c\times n}$ using the membership $u_{ij}^{\left(k\right)}$.10:   Update the centers $c_{i}^{\left(k\right)}$ using $u_{ij}^{\left(k\right)}$11: $c_{i}=\frac{\sum_{j=1}^{n}u_{ij}^{m}x_{j}}{\sum_{j=1}^{n}u_{ij}^{m}}$12:**until** $∥U^{\left(k+1\right)}-U^{\left(k\right)}∥<\epsilon$13:**Return** $c_{i}^{\left(k\right)}$

### 2.2. Mahalanobis-Distance-Based Fuzzy C-Means Clustering

Malignant breast tumours result, in part, from physiological dysfunction [3,17]. They can be irregular, lobular, and ill-defined in ways that may not be captured using Euclidean distance [3,17]. Mahalanobis distance is a dissimilarity metric calculated using a covariance matrix and therefore takes into consideration the variance and correlation of data points. By replacing the Euclidean distance in FCM with the Mahalanobis distance, one enables the fuzzy c-means algorithm to mitigate its limitations as it permits a multivariate approach to breast cancer detection [17]. Ref. [18] replaced Euclidean distance with Mahalanobis distance (FCM-M) to classify arrhythmic beats on electrocardiograms. The proposed FCM-M performed significantly better than base FCM and reduced iterations in the numerical algorithm to an average of 53% of the base FCM.

The Mahalanobis distance is defined as

(5) $$d^{2}\left(x_{j},c_{i}\right)={\left(x_{j}-c_{i}\right)}^{T}\Sigma_{i}^{-1}\left(x_{j}-c_{i}\right)$$

where $\Sigma_{i}$ is the fuzzy covariance matrix. To use the Mahalanobis distance for FCM, we must derive a new set of update functions. As outlined by Hadler [18], our objective function is given by

(6) $$J\left(U,C,\Sigma ;X,m\right)=\sum_{i=1}^{c}\sum_{j=1}^{n}u_{ij}^{m}\left[{\left(x_{j}-c_{i}\right)}^{T}\Sigma_{i}^{-1}\left(x_{j}-c_{i}\right)-ln\left|\Sigma_{i}^{-1}\right|\right]$$

where the same definitions hold for *U* and *C*, constraints (1) and (2) are maintained, and $-ln|\Sigma_{i}^{-1}|$ is a “regulating factor of the covariance matrix” [18]. With this formulation, one must rederive the membership, centroid, and fuzzy covariance matrix update functions. In this case, the Lagrangian is

$$L\left(U,C,\Sigma ,\lambda ;X,m\right)=\sum_{i=1}^{c}\sum_{j=1}^{n}u_{ij}^{m}\left[{\left(x_{j}-c_{i}\right)}^{T}\Sigma_{i}^{-1}\left(x_{j}-c_{i}\right)-ln\left|\Sigma_{i}^{-1}\right|\right]+\sum_{j=1}^{n}\lambda_{j}\left(\sum_{i=1}^{c}u_{ij}-1\right).$$

By solving the optimization problem, we have the membership update function for a specific cluster *k* and datapoint *l*

$$u_{kl}=1/\sum_{i=1}^{c}{\left[\frac{{\left(x_{l}-c_{k}\right)}^{T}\Sigma_{k}^{-1}\left(x_{l}-c_{k}\right)-ln\left|\Sigma_{k}^{-1}\right|}{{\left(x_{l}-c_{i}\right)}^{T}\Sigma_{i}^{-1}\left(x_{l}-c_{i}\right)-ln\left|\Sigma_{i}^{-1}\right|}\right]}^{\frac{1}{m-1}},$$

the centroid update function for a cluster *k*

$$c_{k}=\frac{\sum_{j=1}^{n}u_{kj}^{m}x_{j}}{\sum_{j=1}^{n}u_{kj}^{m}},$$

and the update function for the fuzzy covariance matrix for a cluster *k*

$$\Sigma_{k}=\frac{\sum_{j=1}^{n}u_{kj}^{m}\left(x_{j}-c_{k}\right){\left(x_{j}-c_{k}\right)}^{T}}{\sum_{j=1}^{n}u_{kj}^{m}}.$$

The pseudocode for FCM-M is adapted from [18].

As can be seen in Algorithm 2, the initial clusters play an important role. Arbitrary centroid settings may lead to local solutions or slow convergence rates. In the following subsections, we introduce three centroid initialization algorithms: the firefly algorithm, the genetic algorithm, and the biogeography-based optimization algorithm.
**Algorithm 2** FCM-Mahalanobis Distance1:Initialize the number of clusters *c*, the degree of fuzziness *m*, the convergence error ϵ2:Randomly initialize the membership matrix $U^{\left(k\right)}={\left[u_{ij}\right]}_{c\times n}$ subject to constraints (1) and (2)3:Update the centroids according to $c_{i}=\frac{\sum_{j=1}^{n}u_{ij}^{m}x_{j}}{\sum_{j=1}^{n}u_{ij}^{m}}$ where i=1,2,3,…,c4:Update the fuzzy covariance matrix according to $\Sigma_{i}=\frac{\sum_{j=1}^{n}u_{ij}^{m}\left(x_{j}-c_{i}\right){\left(x_{j}-c_{i}\right)}^{T}}{\sum_{j=1}^{n}u_{ij}^{m}}$5:Update the memberships values according to $\frac{1}{\sum_{l=1}^{c}{\left[\frac{{\left(x_{j}-c_{i}\right)}^{T}\Sigma_{i}^{-1}\left(x_{j}-c_{i}\right)-ln\left|\Sigma_{i}^{-1}\right|}{{\left(x_{j}-c_{l}\right)}^{T}\Sigma_{l}^{-1}\left(x_{j}-c_{l}\right)-ln\left|\Sigma_{l}^{-1}\right|}\right]}^{\frac{1}{m-1}}}$ and store in a matrix $U^{\left(k+1\right)}$  6:If $∥U^{\left(k+1\right)}-U^{\left(k\right)}∥<\epsilon$ stop. Otherwise, continue from 3.

### 2.3. Firefly Algorithm

Developed in 2008 by Xin-She Yang, the firefly algorithm (FA) is an optimization algorithm based on the behaviour of fireflies [19]. FA is based on the following principles:

1. Fireflies are attracted to each other and tend to move towards the brightest one.
2. Fireflies are unisex; thus, fireflies are attracted to one another regardless of sex.
3. The brightness of a firefly is proportional to its attractiveness and inversely proportional to distance. As distance increases, brightness decreases; therefore, the solution is less optimal.
4. Fireflies move randomly, but their movement is biased towards brighter fireflies.

The algorithm represents potential solutions as fireflies, and then with each iteration, updates their position based on their brightness and distance from others [19]. The brightness of the firefly represents how desirable the solution is [19]. Any function may be used as an objective function for brightness; however, for the purposes of initializing FCM, we opt for the sum of squared Euclidean distances between pixel values and fireflies.

The movement of a firefly *i* who is attracted to a brighter firefly *j* is dictated by [19]:

(7) $$x_{i}=x_{i}+\beta_{0}e^{-\gamma r_{i,j}^{2}}\left(x_{j}-x_{i}\right)+\alpha \epsilon_{i}$$

where α is a randomization parameter, $\epsilon_{i}$ is a random vector taken from either the Gaussian or uniform distributions, and $r_{i,j}$ is the Cartesian distance:

(8) $$r_{i,j}=\sqrt{\sum_{k=1}^{d}{\left(x_{i,k}-x_{j,k}\right)}^{2}}$$

β defines the attractiveness of a firefly and is given by:

(9) $$\beta =\beta_{0}e^{-\gamma r^{2}}$$

where $\beta_{0}$ is the attractiveness at r=0 and γ is the light absorption coefficient. The pseudocode based on [19] is shown in Algorithm 3.
**Algorithm 3** Firefly Algorithm1:Objective function f(x), $x={\left(x_{1},\ldots ,x_{d}\right)}^{T}$2:Generate initial population of fireflies $x_{i}$(i=1,2,…,n)3:Light intensity *I* is given by $f(x_{i})$ where f(·) is the chosen objective function4:Define light absorption coefficient γ5:**while** t< maxGeneration **do**6:    **for** 1≤i≤n **do**7:     **for** 1≤j≤n **do**8:     **if** $I_{i}<I_{j}$ **then**9:       Move firefly *i* towards firefly *j*10:      **end if**   Vary attractiveness according to *r*11:   Evaluate new solution and update light intensity12:     **end for**13:    **end for**14:    Rank the fireflies and find the current global best $g_{*}$15:**end while**

### 2.4. Genetic Algorithm

First introduced in 1975 by Holland [20], the genetic algorithm imitates the process of natural selection to determine the best potential solution to the problem [20,21]. It first creates a population of potential solutions to the problem and then uses the three principles characteristic of GA to combine and optimize these solutions [20,21]. The three principles are as follows:

1. Selection, where the fitness of each solution is evaluated and the two best solutions will reproduce.
2. Crossing, where the two solutions with the best potential exchange information to create offspring solutions that are a combination of both the paternal and maternal genetic information.
3. Mutation; some of the offspring undergo random and permanent changes in their genetic information to introduce novel genetic information to the population. Mutations increase the diversity of the gene pool to better explore the search space.

The pseudocode given by [9] is shown in Algorithm 4.
**Algorithm 4** Genetic Algorithm1:Randomly generate a population *P* of n solutions2:**repeat**3:     $p^{\prime }=\emptyset$4:      **repeat**5:        Selection of 2 solutions *x* and $x^{\prime }$ of *P*6:        Crossing between the two parents *x* and $x^{\prime }$ to form two children *y* and $y^{\prime }$7:        Mutate *y* and $y^{\prime }$ under certain conditions8:        Add *y* and $y^{\prime }$ in $P^{\prime }$9:      **until** $\left(|P^{\prime }|=n\right)$10:     $P=P^{\prime }$11:    **until** shutdown criteria are met

It should be noted there are many implementation styles for GA. For our Python package, we opt for roulette wheel selection, single-point crossover, and Gaussian mutation [20,21].

Iterations in GA are run until a satisfactory solution is produced or a stopping criterion is met [21]. GA in breast cancer segmentation is often used to optimize a machine learning model to improve its accuracy. In an effort to detect breast cancer, Ref. [22] combined GA with a neural network (NN) and showed that combining GA with NNs was more effective than traditional NNs. Ref. [23] combined it with mutual information (MI) to select the best combination of cancer predictors where the intersection of the two resulted in highly accurate predictions of breast cancer.

Ref. [13] first proposed FCM using GA to implement the concept of gradation of membership, where one data point belongs to multiple clusters with different membership values. Its performance is comparable to certain NN techniques as the combination of GA and FCM overcomes the risk of getting stuck in local optima [13]. Ref. [24] also supported the use of GA to find the initial clusters for FCM and introduced the fuzzy c-means genetic algorithm (FGA) for segmenting grey-scale images. FGA generated fine and smooth clusters compared to FCM and hard c-means clustering, showing great potential for segmenting complex data [24]. To overcome the local optima, Ref. [14] uses a quantum-inspired GA (QGA) to determine optimal initial clusters for FCM. The proposed hybrid algorithm (QEE-FCM) demonstrated accuracy in segmentation with reduced runtime, offering the user a balance between accuracy and computational effort [14]. Ref. [25] used GA as an initialization algorithm for FCM for fault diagnosis in a satellite attitude determination system (ADS). After 20 iterations, the hybrid algorithm obtained the correct partition with an average objective function of 0.2681 compared to the 0.2755 obtained from FCM. The hybrid algorithm, therefore, resulted in a more optimal partition than FCM.

### 2.5. Biogeography-Based Optimization

Biogeography-based optimization (BBO) refers to a class of algorithms based on biogeography, which studies the patterns of species distribution across habitats [26]. Inspired by the initial biogeography-based algorithm, Simon [26] proposed the metaheuristic algorithm to determine the best possible solution to a given problem. Each solution to the optimization problem is known as a “habitat” [26]. Habitats with greater fitness for the species are known to have a high habitat suitability index (HSI). The factors that make the habitat suitable are termed suitability index variables (SIV). In an optimization problem, HSI represents the fitness value of the solution while SIV is its component. The mathematical model, foundational to BBO, considers the factors that affect species distribution in the wild: migration rate between habitation, extinction rate, and mutation rate of species [26]. Since the goal of an optimization problem is to converge on an optimal solution, Simon [26] theorized that a solution with high HSI will send some of its SIV to a solution with poor HSI; this is termed emigration. A solution with a low HSI will accept SIV from a solution with a high HSI; this is termed immigration. Through this process, BBO improves the fitness of the solutions and selects the best one. Given a maximum immigration rate *I*, maximum emigration rate *E*, and the maximum number of species *N*, the following formulae for the migration process are defined.

The immigration rate with *k* species is given by [9]:

(10) $$\lambda_{k}=I\left(1-\frac{k}{N}\right)$$

The emigration rate with *k* species is given by [9]:

(11) $$\mu_{k}=E\left(\frac{k}{N}\right)$$

The habitat probability is calculated as in [27]:

(12) $$P\left(i\right)=\frac{v_{i}}{\sum_{k=1}^{n}v_{k}}$$

where $v_{i}$ is defined using

(13) $$v_{i}=\left\{\begin{array}{cc} \frac{n!}{(n-1-i)!(i-1)!}, & i=1,\ldots ,\lceil (n+1)/2\rceil \\ v_{n+1-i}, & i=\lceil (n+1)/2\rceil +1,\ldots ,n, \end{array}\right.$$

where ⌈(n+1)/2⌉ is the smallest integer that is greater than or equal to (n+1)/2. The mutation rate of the *i*th habitat is calculated as in [27]:

(14) $$\pi_{i}=\pi_{\max}\left(1-\frac{P(i)}{P_{\max}}\right)$$

The pseudocode is shown in Algorithm 5. Since BBO does not make assumptions about the problem, it can be applied to problems of different natures. Furthermore, Santosa and Safitri [28] state that BBO is good at solving continuous problems. Applications of BBO to breast cancer include predicting breast cancer survival rates based on cancer’s pathological features [29]. Zhang et al. [27] recommended a hybrid BBO and FCM algorithm to overcome FCM’s reliance on initial clusters. Their proposed algorithm uses random initialization to generate the initial clustering solutions and then implements an evolutionary algorithm to find the optimal solution. At the end of the evolutionary algorithm, the FCM algorithm is applied to the best initial clustering solution to segment the data [27]. The proposed algorithm resulted in better partitioning than FCM and achieved clear clusters on test images. However, BBO-FCM is prone to overlapping, as evidenced by small PC values [27].
**Algorithm 5** Biogeography-Based Optimization1:Randomly generate a population *P* of *n* solutions2:**while** the stop criterion is not met **do**3:    Evaluate the HSI of each solution4:    Calculate the number of species *S*, the rate of immigration λ and emigration μ for each solution5:    **for** 1≤i≤n **do** Use $\lambda_{i}$ to decide, in a probabilistic way, to migrate towards a solution *i*6:     **if** rand$\left(0,1\right)<\lambda_{i}$ **then** Replace a randomly chosen variable in solution *i* with the variable in solution *j*7:     **end if**8:    **end for** Mutation: mutating individuals9:**end while**

### 2.6. Cooperation of Metaheuristics

The cooperation of metaheuristics combines two or more algorithms from the class of metaheuristics to overcome the limitations of each algorithm separately [30]. It involves amalgamating the strengths of multiple algorithms to find an optimal solution based on more parameters and restrictions [30]. To combine these algorithms, developers might implement hybridization or parallelization. Hybridization uses the output of one metaheuristic algorithm to inform the search of the other, whereas parallelization runs both algorithms simultaneously to foster the exchange of information between the two. One of the key advantages of combining metaheuristic algorithms is that it minimizes the need to accurately select the algorithm best suited to solve the optimization problem of interest [30]. Tezel and Mert [30] state that, for the best results, the two algorithms selected should compensate for each other’s limitations.

## 3. Image Analysis

We implemented the above fuzzy c-means clustering algorithm and uploaded the optimized Python package on the website: https://github.com/Danyulll/FuzzyPySeg (accessed on 11 April 2023). The FCM function package is user-friendly and requires specification of the fuzziness degree and number of clusters with cluster method in either Euclidean distance or Mahalanobis distance. The other algorithms, such as FA, GA, and BBO, are executed by stating the centroid initialization. Parallel computing was used to decrease the computation time, shown in Table 1, Table 2 and Table 3.

The FCM-based algorithms discussed above were tested using two images. The first is a clustering image containing six shapes. The image has clear borders for each clustering and makes it easy to test the effectiveness of these algorithms. The second image is a digital mammogram with potentially cancerous tumours from the VinDr-Mammo database [31].

**Figure 1:**
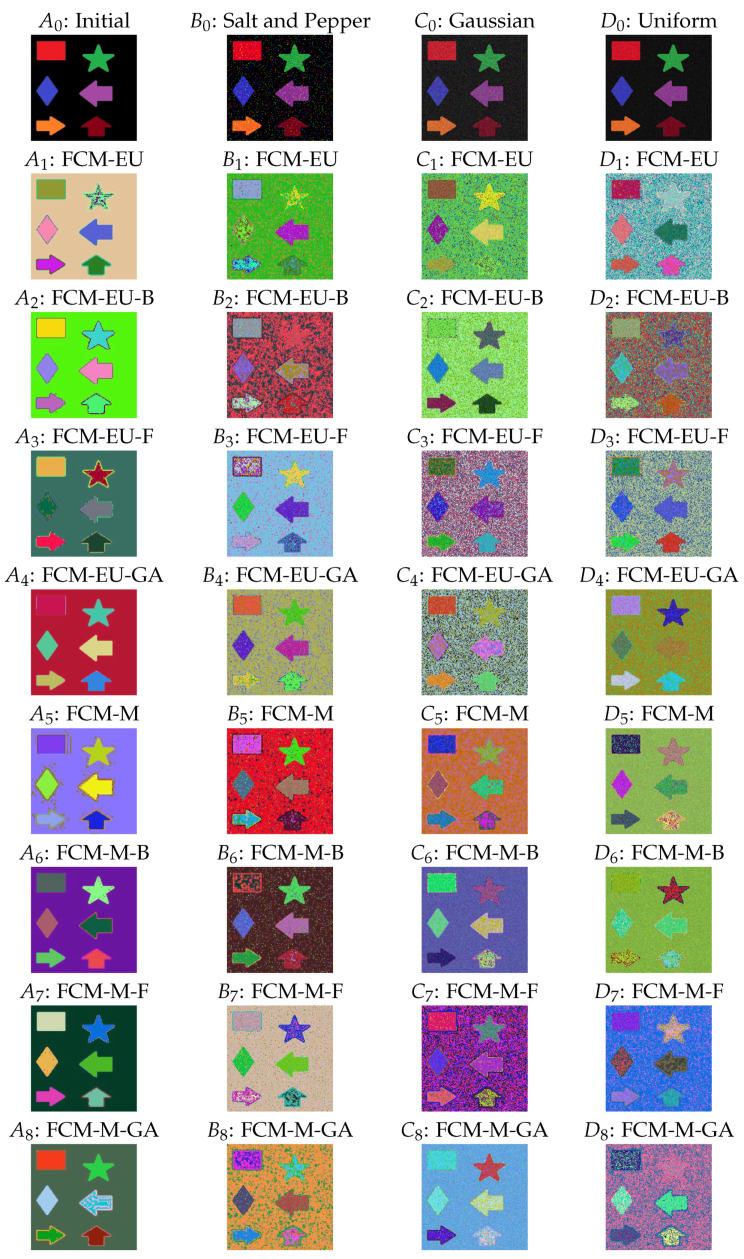
($A_{0}$) Initial image. ($B_{0}$) Salt and pepper noise. ($C_{0}$) Gaussian noise. ($D_{0}$) Uniform noise. ($A_{1}$–$A_{8}$): Euclidean-distance-based fuzzy c-means clustering (FCM-EU), Euclidean-distance-based biogeography-based optimization (FCM-EU-B), Euclidean-distance-based firefly algorithm (FCM-EU-F), Euclidean-distance-based genetic algorithm (FCM-EU-GA), Mahalanobis-distance-based fuzzy c-means clustering (FCM-M), Mahalanobis-distance-based biogeography-based optimization (FCM-M-B), Mahalanobis-distance-based firefly algorithm (FCM-M-F), and Mahalanobis-distance-based genetic algorithm (FCM-M-GA), for ($A_{0}$). Similar for ($B_{1}$–$B_{8}$), ($C_{1}$–$C_{8}$), and ($D_{1}$–$D_{8}$) and for ($B_{0}$), ($C_{0}$), and ($D_{0}$), respectively.

For the clustering image, we set the number of clusters as C=7 and the degree of fuzziness as m=2. We considered three noise images including salt and pepper, Gaussian, and uniform types. In Figure 1, $A_{0}$ is the initial image, and $B_{0}$, $C_{0}$, and $D_{0}$ are the corresponding noise images of the initial image for salt and pepper (with noise density 0.05), Gaussian (with variance 0.01), and uniform noise (with bound [−1,1]) type, respectively. See more information on the type of noise in the R package imgnoise. The four initial images are shown in the first row, and their clustering effect under different algorithms is plotted and presented in the following eight rows.

From the images under different algorithms, we can observe:

1. As observed in Figure 1($A_{5}$), arbitrary centroid settings resulted in imperfect segmentation by Mahalanobis-distance-based fuzzy c-means clustering (FCM-M) as there are random dots around the edges of the shapes. The three centroid initialization algorithms worked well in segmenting the edges of different shapes, as shown in ($A_{6}$–$A_{8}$).
2. Mahalanobis distance methods perform better than Euclidean distance methods. For example, consider the shape star in ($A_{1}$); the Euclidean-distance-based fuzzy c-means clustering (FCM-EU) contained two colours and was not clustered well, whereas in ($A_{5}$), the star shape had only one colour and all other shapes were classified effectively. A similar outcome is noted for the star and rhombus in ($A_{3}$) by the Euclidean-distance-based firefly algorithm (FCM-EU-F) and ($A_{7}$) by the Mahalanobis-distance-based firefly algorithm (FCM-M-F). A clearer clustering was obtained when using FCM-M.
3. In practice, images may contain noise that influences the accuracy and quality of the image segmentation. When different types of noise were applied to the images, as seen in columns 2, 3, and 4 in Figure 1, the algorithms determined the basic clustering for the edges of different shapes. However, the accuracy was reduced, as evidenced by the presence of two colours in the background in ($B_{8}$: FCM-M-GA). Nonetheless, FCM-M outperforms FCM-EU in most scenarios. For example, some shapes in ($B_{1}$–$B_{4}$) under Euclidean distance have unclear edges, but the same edges are clear in ($B_{5}$–$B_{8}$) under Mahalanobis distance. This trend is observed in the Gaussian and uniform noise images as well, as ($C_{1}$) and ($C_{4}$) have many colourful dots in the background, but in ($C_{5}$) and ($C_{8}$), the background is more smooth.

**Figure 2:**
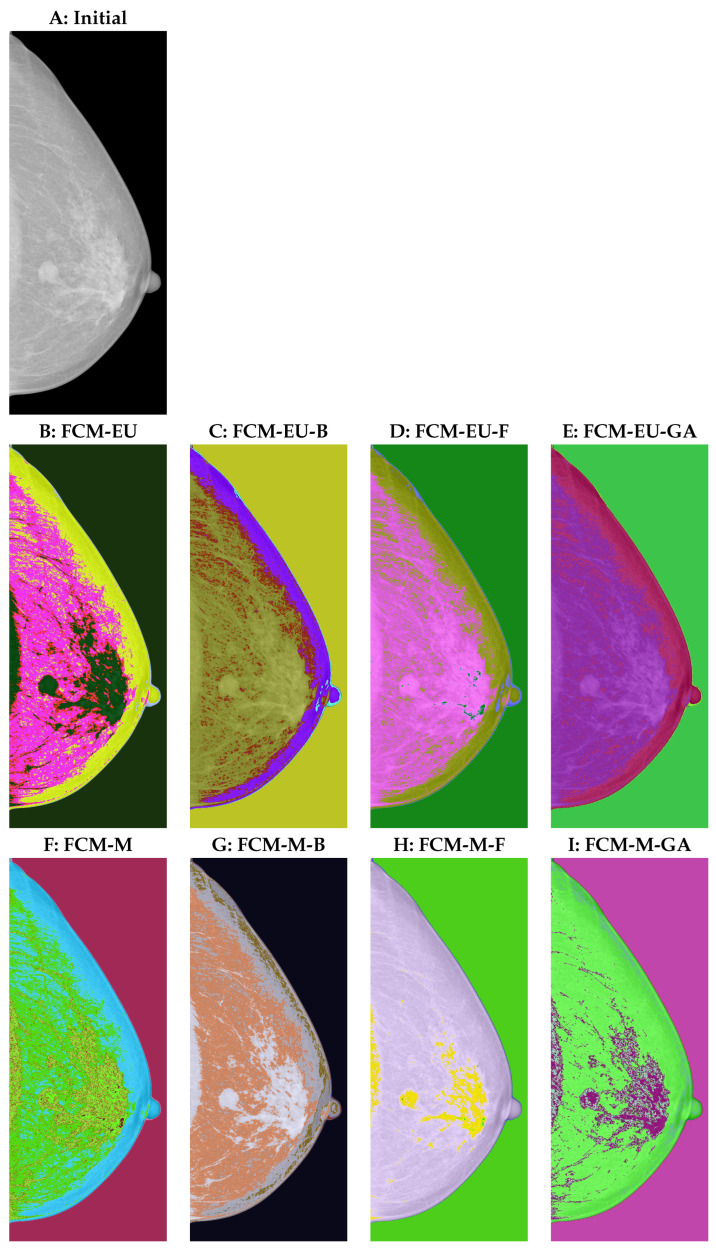
(**A**) Initial image. (**B**) Euclidean-distance-based fuzzy c-means clustering (FCM-EU). (**C**) Euclidean-distance-based biogeography-based optimization (FCM-EU-B). (**D**) Euclidean-distance-based firefly algorithm (FCM-EU-F). (**E**) Euclidean-distance-based genetic algorithm (FCM-EU-GA). (**F**) Mahalanobis-distance-based fuzzy c-means clustering (FCM-M). (**G**) Mahalanobis-distance-based biogeography-based optimization (FCM-M-B). (**H**) Mahalanobis-distance-based firefly algorithm (FCM-M-F). (**I**) Mahalanobis-distance-based genetic algorithm (FCM-M-GA).

We then applied the above algorithms to a mammogram in order to evaluate its performance in breast cancer detection. In the initial image, Figure 2A, the tumour region varies little from the surrounding area. However, locating the areas of greater density and using the information from the VinDr-Mammo database, we confirmed the tumour area [31]. The area detected by human eyes should be confirmed by optimized segmentation algorithms using the same degree of fuzziness m=2 and the number of clusters C=3. As we can see from Figure 2,

1. The three centroid initialization algorithms resulted in clearer clustering groups than the arbitrary centroid settings. As shown in the second row in Figure 2, C(FCM-EU-B), D(FCM-EU-F), and E(FCM-EU-GA) better captured the white regions than B (FCM-EU), making the image clustering more accurate. Similarly, G (FCM-M-B), H (FCM-M-F), and I (FCM-M-GA) captured the tumour region with clearer segmentation than F (FCM-M).
2. FCM-M resulted in a higher-quality segmentation of the tumour region from its surroundings than FCM-EU. Comparing D to the Euclidean-distance-based firefly algorithm (FCM-EU-F) and H to the Mahalanobis-distance-based firefly algorithm (FCM-M-F), we found that FCM-M-F can clearly locate the tumour region with the help of additional spots in a different colour, and hence it highlights the region with greater clarity than FCM-EU-F.
3. A similar observation is made for non-tumour regions. FCM-M classified the details of the image better than FCM-EU. The implementation of centroid initialization algorithms further improved the quality of the segmentation. For example, the nipples in Figure 2B–E were classified with two colours; however, human eyes detect that there should not be two colours in that area. Nipples are clustered well in H and I with only one colour. In addition, the upper edge of the breast should not have been identified as separate from other tissues in the breast. This works well in H and I. However, in Figure 2B–E, the Euclidean-distance-based function segmented them separately. In Figure 2F, with the arbitrary centroid settings, the upper edge of the breast was separated from the other breast tissue, as supported by the different colours. This is improved in H and I when using the firefly and genetic algorithms.

## 4. Conclusions and Limitations

Several studies have highlighted the potential of fuzzy c-means clustering (FCM) in breast cancer image segmentation [8,9]. However, FCM has notable drawbacks in determining optimal initialization and the number of clusters. This review paper explores FCM with respect to Mahalanobis distance and Euclidean distance functions in addition to three promising initialization algorithms that address the limitations of base FCM. We compared the different FCM algorithms using two images, assessing the quality of segmentation in each. Combining one of the three centroid initialization algorithms with basic FCM enhanced the quality of the segmentation significantly. Mahalanobis-distance-based FCM produced images with higher clustering accuracy than the Euclidean-distance-based FCM. The difference in accuracy may be acquitted to the consideration of correlation in data in the Mahalanobis distance function. The image analysis suggests that the discussed algorithms show potential for the computer-aided segmentation of breast tumours in medical imaging. Future research will address several limitations of the above algorithms. In particular, we will focus on quantifying the accuracy of the segmentation using metrics, improving their robustness in images with noise, and reducing computational time.

## Figures and Tables

**Table 1:** Comparison of the run times of the FCM Mahalanobis algorithm before and after optimization on the BIRAD 2 image over 3 iterations.

	Before Optimization	After Optimization
Test Number	Run Time	Run Time
1	73 m 51 s	2 m 21 s
2	72 m 39 s	2 m 20 s
3	73 m 16 s	2 m 19 s
Average Time	73 m 15 s	2 m 20 s
Average Time Per Iteration	24 m 25 s	46.6 s

**Table 2:** Comparison of the run times of the firefly algorithm before and after optimization on the Birad 2 image over 5 iterations with a population size of 20.

	Before Optimization	After Optimization
Test Number	Run Time	Run Time
1	1412 m 18 s	17.0 s
2	1410 m 54 s	17.1 s
3	1415 m 23 s	16.9 s
Average Time	1412 m 52 s	17.0 s
Average Time Per Iteration	470 m 57 s	3.4 s

**Table 3:** Comparison of the run times of the BBO algorithm before and after optimization on the BIRAD 2 image over 20 iterations with a population size of 50.

	Before Optimization	After Optimization
Test Number	Run Time	Run Time
1	462 m 1 s	21.7 s
2	459 m 16 s	21.8 s
3	461 m 46 s	21.7 s
Average Time	461 m 1 s	21.7 s
Average Time Per Iteration	23m 3 s	1.1 s

## Data Availability

The breast cancer image can be accessed from https://physionet.org/content/vindr-mammo/1.0.0/, accessed on 15 February 2023.
